# Exploring the Toxin-Mediated Mechanisms in *Clostridioides difficile* Infection

**DOI:** 10.3390/microorganisms12051004

**Published:** 2024-05-16

**Authors:** Evdokia Pourliotopoulou, Theodoros Karampatakis, Melania Kachrimanidou

**Affiliations:** 1 Department of Microbiology, Medical School, Aristotle University of Thessaloniki, 541 24 Thessaloniki, Greece; evapourlio6@gmail.com; 2 Microbiology Department, Papanikolaou General Hospital, 570 10 Thessaloniki, Greece; tkarampatakis@yahoo.com

**Keywords:** *Clostridioides difficile*, infection, pathogenesis, bacterial toxins, inflammation, actin cytoskeleton

## Abstract

*Clostridioides difficile* infection (CDI) is the leading cause of nosocomial antibiotic-associated diarrhea, and colitis, with increasing incidence and healthcare costs. Its pathogenesis is primarily driven by toxins produced by the bacterium *C. difficile*, Toxin A (TcdA) and Toxin B (TcdB). Certain strains produce an additional toxin, the *C. difficile* transferase (CDT), which further enhances the virulence and pathogenicity of *C. difficile*. These toxins disrupt colonic epithelial barrier integrity, and induce inflammation and cellular damage, leading to CDI symptoms. Significant progress has been made in the past decade in elucidating the molecular mechanisms of TcdA, TcdB, and CDT, which provide insights into the management of CDI and the future development of novel treatment strategies based on anti-toxin therapies. While antibiotics are common treatments, high recurrence rates necessitate alternative therapies. Bezlotoxumab, targeting TcdB, is the only available anti-toxin, yet limitations persist, prompting ongoing research. This review highlights the current knowledge of the structure and mechanism of action of *C. difficile* toxins and their role in disease. By comprehensively describing the toxin-mediated mechanisms, this review provides insights for the future development of novel treatment strategies and the management of CDI.

## 1. Introduction

*Clostridioides difficile*, commonly known as *C. difficile*, is a Gram-positive obligate anaerobic bacterium that produces toxins [1]. It exists either in vegetative form or in highly resistant spore form. Spores can be found in the environment and food and represent the infectious form of *C. difficile*, while vegetative cells cannot survive outside the host’s anaerobic environment. Transmission occurs via the fecal–oral route, with both forms capable of infecting the host; however, only spores can survive the acidic stomach environment, allowing them to colonize the intestine, where they proliferate and produce the key virulence factors, Toxin A (TcdA) and Toxin B (TcdB) [2]. These toxins disrupt the integrity of the intestinal epithelium, leading to tissue damage, inflammation, and diarrhea. Additionally, the supplementary *C. difficile* transferase (CDT) enhances virulence, collectively contributing to the pathogenesis of *C. difficile* infection (CDI) [3,4].

The epidemic strain BI/NAP1/027 produces elevated levels of TcdA and TcdB, exhibiting high fluoroquinolone resistance and is associated with increased morbidity and mortality [5,6]. This hypervirulent strain’s increased toxin production, particularly during both exponential and stationary growth phases, correlates with its high spore counts, enabling it to outcompete other strains in diverse environments, enhancing colonization and disease severity [7,8]. Additionally, it encodes a variant of TcdB, which is cytotoxic across various cell lines, further contributing to its virulence [9]. Moreover, the production of CDT enhances its colonization ability and the severity of illness [10,11].

*C. difficile* typing is crucial for epidemiological surveillance and understanding the diversity of this bacterium. Molecular typing methods such as PCR-ribotyping, pulsed-field gel electrophoresis (PFGE), multilocus variable-number tandem repeat analysis (MLVA), multilocus sequence typing (MLST), and toxinotyping allow the characterization of strains based on genetic variations [12,13,14]. These methods provide valuable information on strain relatedness, transmission routes, and outbreak investigations, aiding in the management and prevention of CDI. By identifying specific genetic markers and profiles, researchers can track the spread of *C. difficile* strains within healthcare settings and communities, facilitating targeted control measures [15,16].

CDI presents with a spectrum of clinical manifestations from mild diarrhea to severe pseudomembranous colitis, toxic megacolon, and colonic perforation [17,18,19]. Various risk factors contribute to CDI, including the administration of broad-spectrum antimicrobials, advanced age, comorbidities, proton pump inhibitor use, prior gastrointestinal surgery, and prolonged hospitalization [20,21]. *C. difficile* is classified by the Centers for Disease Control and Prevention (CDC) as one of the top five urgent threats to human health [22]. Historically healthcare-associated, CDI shows an alarming rise in community-acquired cases with approximately 780,000 infections and 49,000 deaths annually in Europe and the United States. These data emphasize the significance of CDI as a public health problem, compounded by its economic burden, exceeding USD 3 billion in Europe and USD 800 million in the United States annually [23,24]. Managing and treating CDI is challenged by the pathogen resistance, easy transmission, and high rates of recurrence rates (20–25% post-treatment), often necessitating alternative therapies [25,26,27]. Additionally, the global spread of the hypervirulent BI/NAP1/027 strain has altered the epidemiology of *C. difficile* [7].

This review focuses on recent advances in understanding how TcdA, TcdB, and CDT interact with host cells, altering the cellular physiology and immune responses. We unravel their binding mechanisms to cellular receptors and manipulation of intracellular signaling pathways, shedding light on the intricate toxin–host dynamics. These toxins are pivotal in CDI pathogenesis, disrupting cell adhesion, and cytoskeletal rearrangements, and triggering pro-inflammatory responses and cell death. Moreover, this review provides a brief overview of potential toxin-based therapeutic strategies, presenting promising approaches for novel CDI treatments.

## 2. TcdA and TcdB

The main virulence factors of *C. difficile* are TcdA and TcdB, belonging to the family of large clostridial toxins (LCTs), which also includes the hemorrhagic toxin (TcsH) and lethal toxin (TcsL) of *Clostridium sordellii*, the large toxin (TpeL) of *Clostridium perfringens*, and the alpha-toxin (TcnA) of *Clostridium novyi* [28,29]. TcdA and TcdB are glucosyltransferases that irreversibly modify the Rho and Ras enzymes associated with guanosine triphosphate (GTPases) [30]. The inactivation of regulatory GTPases results in the destruction of the actin cytoskeleton, cell rounding, and ultimately cell detachment [28,31]. TcdA was termed an enterotoxin due to its ability to induce enterotoxicity in animals, characterized by inflammation, cytokine release, and fluid secretion, while also disrupting tight junctions (TJs) in human intestinal epithelial cells [32,33]. In contrast, TcdB, though not inducing similar symptoms, was 100 to 1000 times more toxic than TcdA in most cell cultures, and it was classified as a cytotoxin. However, experiments in mice with human intestinal transplants revealed that TcdB is equally potent as an enterotoxin, causing epithelial cell damage, acute inflammation, and increased mucosal permeability [34,35].

The production of TcdA and TcdB are essential for the pathogenesis of CDI, while TcdB plays a crucial role, as evidenced by strains producing only the TcdB inducing all CDI symptoms, sometimes more severely than strains producing both TcdA and TcdB [36,37]. Initially, TcdA was considered the primary virulence factor, but subsequent studies showed that both toxins contribute synergistically to disease progression [38,39]. While TcdA was thought to enhance the action of TcdB, strains solely producing TcdB exhibited significant virulence, challenging the notion of TcdA’s primary role [40,41]. Recent findings indicate that TcdB is more closely associated with CDI severity, supported by clinical strains predominantly producing TcdB and strains lacking TcdA but causing severe disease [36,41]. These insights highlight the intricate interplay of TcdB as an emerging key determinant of disease severity.

### 2.1. Genetics and Structure of TcdA and TcdB

**Figure 1:**
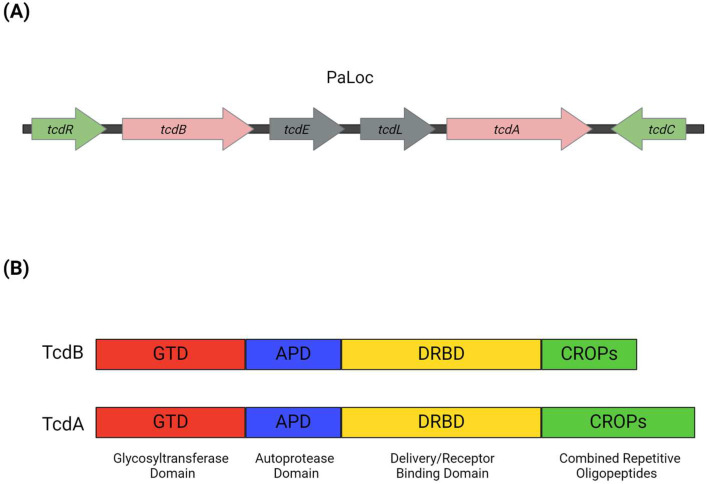
(**A**) Pathogenicity Locus (PaLoc). The genes *tcdA* and *tcdB* (pink arrows) encode the toxins TcdA and TcdB, respectively. The regulatory genes *tcdR* (positive) and *tcdC* (negative) modulate the transcription of *tcdA* and *tcdB* and are presented with green arrows. Genes *tcdE* and *tcdL* (grey arrows) encode a holin and an endolysin, respectively, which are involved in toxin secretion. The direction of the arrows represents the direction of transcription of the genes. (**B**) TcdA and TcdB are divided into four domains: the glycosyltransferase domain (GTD; red), the autoprotease domain (APD; blue), the delivery and receptor-binding domain (DRBD; yellow), and the combined repetitive oligopeptides (CROPs; green).

The genes encoding TcdA (*tcdA*) and TcdB (*tcdB*) are located within the pathogenicity locus (PaLoc) of a 19.6 kb region, which is only found at the same genomic position in toxigenic strains of *C. difficile* [40,42]. PaLoc contains four additional open reading frames including the genes *tcdR*, *tcdE*, *tcdC*, and *tcdL* [43] (Figure 1). The *tcdR* gene encodes the 22 kDa protein TcdR, which is an alternative sigma factor, a member of the σ^70^ family, that positively regulates the transcription of the *tcdA* and *tcdB* genes [44,45]. The *tcdC* gene encodes the protein TcdC with a molecular weight of 26 kDa [46,47]. TcdC acts as an anti-sigma factor negatively regulating the transcription of *tcdA* and *tcdB* [48,49,50]. Various studies have linked the deletion of the *tcdC* gene with increased pathogenicity [37,51]. In the BI/NAP1/027 strain, the deletion of the *tcdC* gene has been implicated in the production of elevated levels of TcdA and TcdB [52,53]. The *tcdE* gene encodes the TcdE protein, which is a member of the class I family of holins and is believed to assist in toxin secretion [45,54]. Analysis of the PaLoc genome revealed the *tcdL* gene, which encodes the TcdL protein, an endolysin that interacts with TcdB and could be involved in toxin secretion [55,56].

TcdA and TcdB are single-chain proteins with molecular weights of 308 kDa and 270 kDa, respectively, and they exhibit a high degree of homology [57,58]. These toxins consist of four conserved functional domains [45]. Upon entry into the host cell, the functional domains of the toxins are activated to complete the infection process [59]. At the N-terminal end, there is the glycosyltransferase domain (GTD), which is responsible for inactivating members of the Rho GTPase family by transferring glucose molecules. The toxins contain domains that help them interact with host cells. These include the autoprotease domain (APD), responsible for autoproteolytic cleavage and toxin processing, and the delivery and receptor-binding domain (DRBD), which is involved in releasing the GTD from intracellular vesicles into the host cell cytoplasm and binding the toxin to cell surface receptors [60,61]. Finally, the C-terminal combined repetitive oligopeptides (CROPs) domain, located at the C-terminal end and composed of five groups of CROPs, also contributes to toxins binding to cell surface receptors [62] (Figure 1).

### 2.2. Mode of Action of TcdA and TcdB

**Figure 2:**
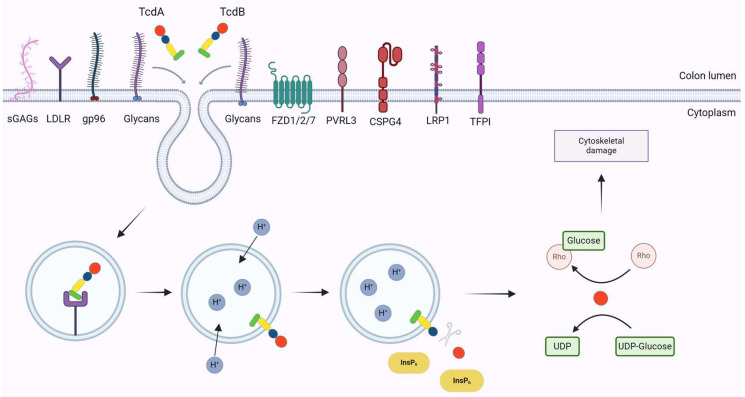
Mechanism of action of TcdA and TcdB. Toxins bind surface receptors on the colonic epithelium and are endocytosed in acidic endosomes. Low pH triggers a conformational change in the toxins resulting in pore formation and translocation of GTD and APD in the cytosol. The activation of APD results in the cleavage and release of the GTD. The GTD blocks the function of Rho and Ras GTPases by transferring the UDP-glycose to GTPases, resulting in the induction of cytoskeletal damage.

The endocytosis and release of TcdA and TcdB into the cytosol of the host cell can be distinguished into five stages: (i) binding of the toxins to cell surface receptors; (ii) cellular uptake via endocytosis; (iii) formation of pores in endosomal membrane; (iv) translocation of the toxin into the cytosol; (v) glycosylation of Rho/Ras GTPases; and (vi) cellular impacts (Figure 2).

#### 2.2.1. Binding to Cellular Receptors

Both TcdA and TcdB interact with various human cell surface glycans via the CROPs domain. Initially, TcdA was reported to bind to sucrase-isomaltase enzyme sites in rabbit ileum, later identifying glycoprotein 96 (gp96) as an additional receptor on colonic epithelium [63]. Sulfated glycosaminoglycans (sGAGs) and low-density lipoprotein receptors (LDLRs) were also recognized as TcdA mediators. LDLR cooperates with sGAGs, facilitating TcdA binding and entry into host cells [64,65,66]. TcdA likely binds to multiple receptors simultaneously for high-affinity cell entry, but further confirmation is required [43].

Although structurally similar, TcdA and TcdB bind to distinct receptors. TcdB’s first identified receptor is chondroitin sulfate proteoglycan 4 (CSPG4), expressed in the subepithelial layer of the intestine [58]. Recently detected in epithelial cell junctions, CSPG4 may represent a soluble form shed by subepithelial fibroblasts [67]. Its Repeat 1 region interacts with TcdB, particularly with the CROPs domain and other regions [68,69]. TcdB also interacts with Frizzled receptors (FZDs), particularly FZD1, 2, and 7, key receptors in the colon epithelium [70,71]. TcdB binds to FZD receptors via a region in the DRBD domain and exploits an endogenous fatty acid as a co-receptor, enhancing specificity and affinity [68]. FZDs are crucial in the Wnt signaling pathway, regulating stem cell proliferation and self-renewal for colon epithelium formation [72]. TcdB binding to FZD receptors disrupts this regulation, affecting cell fate control, proliferation, and differentiation [73].

Another identified receptor is Poliovirus Receptor-Like 3 (PVRL3), highly expressed on colon epithelial cell surfaces. TcdB interacts with PVRL3 outside the CROPs domain [74]. Binding sites for CSPG4 and FZDs on TcdB are separate, potentially allowing simultaneous binding if expressed on the same cell surface, suggesting a “dual-receptor” model [68,75]. CSPG4 is mainly expressed in subepithelial myofibroblasts, while PVRL3 and FZDs are in the intestinal epithelium, serving as independent receptors on different cell types [70]. TcdB may initially bind to PVRL3 and FZDs to enter the colon epithelium and later access CSPG4 in subepithelial myofibroblasts, leading to further mucosal destruction [76]. Protein 1 associated with low-density lipoprotein receptor-related protein 1 (LRP1) is a novel receptor for TcdB, interacting through the CROPs domain and undergoing endocytosis. LRP1, part of the LDL receptor family, is expressed in various cell lines and in the colon epithelial cells in vivo [64].

Variants TcdB2, TcdB4, TcdB10, and a subset of TcdB7 utilize tissue factor pathway inhibitor (TFPI) as a receptor [77]. TFPI is highly expressed in the intestinal epithelium and other types of intestinal cells. TcdB variants exhibit differences in receptor preference based on sequence divergence, with TcdB1-4 being prevalent in epidemic strains. TcdB1 selectively binds to CSPG4 and FZDs, TcdB2 to CSPG4 and TFPI, TcdB3 to FZDs, and TcdB4 to TFPI [78,79]. Differential preference of TcdB variants for cellular receptors is a possible explanation for the differences in the virulence of the variants. Specifically, TcdB of the epidemic strain BI/NAP1/027 (TcdB_NAP1_) exhibits increased cytotoxicity in vitro by altering specificity for cell surface receptors. Comparison of TcdB_NAP1_ with TcdB from the reference strain VPI10463, which is less cytotoxic than TcdB_NAP1_, reveals that the two variants use different cellular receptors, indicating that this difference may account for the increased virulence of TcdB_NAP1_ in vivo [80,81].

#### 2.2.2. Cellular Uptake

Cellular uptake of the toxins occurs immediately after binding to the receptors, as the toxins are endocytosed into host cells via endosomes [40,45]. The endocytosis of TcdB relies on clathrin, while that of TcdA depends on protein kinase C and the substrate for casein kinase 2 (PACSIN2). Clathrin and PACSIN2 are proteins that form the coat of endocytic vesicles, facilitating the endocytosis of a spectrum of transmembrane receptors and their ligands. Subsequently, the newly formed vesicles are removed from the plasma membrane through the action of dynamin. Dynamin is a GTPase that facilitates the cutting of newly formed endocytic vesicles from the plasma membrane and their release into the cytoplasm [82]. Inhibiting the function or expression of dynamin can prevent the entry of toxins TcdA and TcdB into cells and suppress the resulting cellular effects [82,83].

#### 2.2.3. Pore Formation

Once the toxins are endocytosed, they traffic within the cytoplasm with the help of endosomes, which mature and cause a decrease in pH [84]. The drop in pH within endosomes is crucial for the intracellular trafficking of the toxins, a fact confirmed in studies that inhibited the cytotoxic effects of the toxins using lysosomotropic agents [85]. The low pH within endosomes induces a conformational change in the toxins, resulting in the insertion of their hydrophobic regions into the endosomal membranes and the subsequent formation of a pore-like α-helical structure [86,87]. In contrast to TcdB, the formation of pores by TcdA requires membranes enriched in cholesterol [88]. It is worth noting that TcdB from hypervirulent strains of *C. difficile* can be translocated into the cytoplasm more rapidly than toxins from other *C. difficile* strains, due to its ability to undergo conformational changes at a higher pH and thus at an earlier stage of endocytosis. This ability is the result of a change in the sequence of the toxin’s hydrophobic region, allowing for enhanced translocation ability across the endosomal membrane [89].

#### 2.2.4. Translocation and Autoprocessing

Following pore formation, the GTD and APD domains unfold and translocate into the host cytosol [90]. Currently, it is not clear how the toxins relocate their catalytic domains to the host cytosol [91]. Simultaneously or immediately after translocation into the cytosol, the two domains undergo refolding with the assistance of the tailless complex polypeptide 1 ring complex/chaperonin containing tailless complex polypeptide 1 (TRiC/CCT) chaperonin system to attain their biological activity [88]. Subsequently, inositol hexakisphosphate (InsP6) activates the APD, leading to autoprocessing [92]. Autoprocessing occurs after a highly conserved residue, Leu542 in TcdA and Leu543 in TcdB, situated between the APD and GTD [93]. This cleavage results in the detachment of the GTD from the rest of the protein and its release into the cytosol [94]. Although both toxins function via the same mechanism, TcdB is more sensitive to autoproteolytic cleavage induced by InsP6 compared to TcdA [43].

#### 2.2.5. Glycosylation of Rho/Ras GTPases

Once GTD is released into the cytosol, it selectively transfers UDP-glucose to Rho and Ras proteins, leading to their inactivation [92]. Primary targets of glycosylation include RhoA, B, C, Rac1, 2, Cdc42, and isoforms of the Rho family such as RhoG and TC10. Secondary substrates comprise certain Ras proteins such as Ral, Ras, and Rap1, 2 [95,96,97]. The functions of Rho and Ras GTPases exhibit distinct differences, as Rho GTPases are the main regulators of the actin cytoskeleton, while Ras primarily controls cell differentiation and proliferation, angiogenesis, and cell adhesion [30,98]. Operating as molecular switches, Rho proteins activate signaling cascades in response to various environmental signals, resulting in changes in the actin cytoskeleton [99].

The Rho proteins undergo a cycle of activation and inactivation, alternating between an inactive state bound to Guanosine diphosphate (GDP) and an active state bound to Guanosine-5′-triphosphate (GTP) [100]. Glycosylation occurs on a conserved threonine residue (Thr35 in Rac1 and Cdc42, Thr37 in Rho, and Thr61 in R-Ras), which is involved in coordinating the Mg^2+^ ion necessary for GTP binding [101,102]. This residue, located in the switch I region of the Rho proteins, undergoes conformational changes upon GTP binding, affecting protein interactions with effectors and other regulatory proteins involved in signal transduction. Glycosylation retains GTPases in their inactive form, disrupting a series of cellular pathways [37].

Variants of TcdB from different strains of *C. difficile* show distinct selectivity toward Rho/Ras GTPases [103]. For example, the TcdB from strains UK1 (RT 027) and VP10463 modifies RhoA, Rac1, and Cdc42, but not R-Ras. In contrast, the TcdB from strains 8864, NAP1V, VPI1470, and M68 (RT017) modifies R-Ras, Rac1, and Cdc42, but not RhoA. The different preferences for GTPases by variant TcdB may have significant implications for the pathogenicity caused by different *C. difficile* strains in human and animal models [104,105].

#### 2.2.6. Cellular Impacts

The induction of glycosylation by toxins on Rho/Ras proteins, followed by the redistribution of the actin cytoskeleton, results in dramatic changes in cell morphology [106,107]. These disruptions include the loss of stress fiber formation, the rounding of cells referred to as the cytopathic effect (CPE), and the contraction of the cellular body leading to the formation of irregular structures [96,108]. Many have attributed CPE to the inactivation of RhoA. Later, glycosylation of Rac was found to be particularly significant for the cytopathological effects of TcdA and TcdB. Cells producing a modified form of Rac1 that was resistant to glycosylation were protected from the cytopathogenic action of TcdB [109].

The deactivation of Rho GTPases also disturbs the cell cycle progression. Inactivation of RhoA leads to binucleated cell formation by impeding contractile ring formation during cytokinesis [105]. Inactive Rac-1 GTPase delays entry into mitotic phase G2-M by failing to activate the cyclin-dependent kinase 1 (CDK1)/cyclin B complex and Aurora A kinase. Both toxins can reduce the expression of cyclin D1 resulting in the cycle arrest in the G1-S phase [110]. Additionally, TcdA activates p53 and p21 and prevents the G2 to M transition, inducing cell arrest [111].

Inactivated Rho proteins disrupt cell–cell contact, increasing epithelial permeability [112]. TJs maintain barrier function, composed of proteins like occludin and claudin, which interact with F-actin via Zonula occludens (ZO) proteins. TcdA and TcdB disrupt actin, compromising occludin–ZO interaction, TJ integrity, and enhancing paracellular permeability [113,114]. Notably, TcdA particularly disrupts epithelial barriers more than TcdB in HIO cell cultures [115]. Epithelial cells typically prevent bacterial colonization through their polarity and TJs [116]. However, TcdA disrupts these defenses, enabling bacteria to penetrate deeper tissues. Consequently, even low toxin levels can facilitate bacterial colonization in CDI, potentially explaining increased adherence observed in animal models when toxins are present [117].

Damage to the epithelium during CDI requires restoration for intestinal integrity and to prevent recurrence. TcdA and TcdB impede epithelial renewal by inhibiting the Wnt/β-catenin pathway, crucial for intestinal turnover [118]. TcdB binds to the FZD-7 receptor, blocking its activation from Wnt3a and stem cell function, impairing epithelial repair [119,120]. Epidemic ribotype 027 TcdB disrupts stem cell function without FZD receptor binding [118]. TcdA inhibits the Wnt pathway by inactivating Rac1, hindering β-catenin translocation and cell proliferation gene expression [119]. In addition to the Wnt pathway, toxins impact the Hippo pathway, essential for tissue homeostasis and regeneration. TcdA and TcdB degrade key effectors YAP and TAZ, which promote cell proliferation and stem cell renewal. This degradation and inactivation in epithelial cells contribute to intestinal epithelium damage caused by TcdA and TcdB [121]. CDI-induced cellular damage progresses from colon cells to deeper layers of the intestinal mucosa, affecting the enteric glial cells (EGCs), pivotal in gastrointestinal physiology. TcdB induces CPE and causes the senescence of EGCs, which has been reported in various pathological conditions of the gastrointestinal system associated with CDI, such as inflammatory diseases, colorectal cancer, and irritable bowel syndrome. Senescent EGCs exhibit a flattened morphology and undergo early DNA damage and irreversible cessation of the cell cycle in the G1 and G2 phases. Exit from the cell cycle is caused by early expression and upregulation of p27, inactivation of cyclin B1, inactivation of CDK1, leading to inactivation of the CDK1/cyclin B1 complex, and accumulation of the inactive form of CDK1. After exposure to TcdB, the impaired function of surviving EGCs has profound effects on their normal and pathological roles [122,123,124,125].

The diarrhea caused by CDI is characterized by increased secretion and/or decreased absorption in the gastrointestinal tract. The exchangers SLC9A3 (solute carrier family 9 member A3), also known as sodium-hydrogen exchanger 3 (NHE3), and SLC26A3 (solute carrier family 26 member 3), known as down-regulated in adenoma (DRA), are key ion transporters in the apical region of epithelial cells of the lower gastrointestinal tract [126,127]. Prolonged TcdA and TcdB exposure significantly decreases NHE3 and DRA levels, leading to dysfunctional water and solute absorption, causing osmotic diarrhea. Currently, it is unknown whether the toxins affect the transcription of the genes encoding these proteins or if they act at a post-transcriptional level. The toxins could reduce NHE3 and DRA concentration by disrupting the actin cytoskeleton via Rho GTPase inactivation, hindering NHE3 and DRA transport to the apical region and reducing gene expression [128]. Alternatively, post-transcriptional regulation, possibly involving protein degradation via the ubiquitin/proteasome pathway, may occur [127].

TcdA and TcdB induce cell death (referred to as cytotoxic effect) in various cell types such as epithelial and endothelial cells, monocytes, lymphocytes, and neurons of the enteric nervous system within 18–48 h post-exposure [76,116,129]. The deactivation of Rho GTPases leads to apoptosis, which occurs after the appearance of CPE [116]. TcdA and TcdB activate caspase-dependent apoptosis via death receptors or the mitochondrial pathway [130,131]. Both pathways activate caspases 3, 6, and 7, which cleave cytoskeletal proteins and activate a few nucleases, resulting in nuclear fragmentation [132]. In the mitochondrial-dependent pathway, TcdA and TcdB increase mitochondrial outer membrane permeability (MOMP), releasing cytochrome c and activating caspase 9 [129,133]. Changes in MOMP are regulated by the balance of pro-apoptotic and anti-apoptotic proteins of the B-cell lymphoma 2 (Bcl-2) family. Pro-apoptotic proteins such as Bax and Bak promote apoptosis, while anti-apoptotic proteins such as Bcl-2 and Bcl-XL suppress it [131,134]. TcdB decreases Bcl-2 levels and disrupts mitochondrial membrane polarity, releasing pro-apoptotic proteins [135]. TcdA may induce Bak protein production via prostaglandin E2 [136,137]. The death receptor pathway involves caspase 8 activation by the tumor necrosis factor-alpha (TNF-α) or Fas binding, triggering cell death and cytochrome c release from mitochondria [133,138].

TcdB induces epithelial cell death via a bimodal mechanism dependent on toxin concentration [139]. At low concentrations of TcdB, apoptosis is triggered by autoproteolysis and glucosyltransferase action, while concentrations above 100 pM lead to glucosylation-independent cell necrosis [140]. This necrotic or pyknotic mechanism causes rapid death in human intestinal cell cultures and pig intestinal graft models, characterized by mitochondrial swelling and loss of plasma membrane integrity, resulting in cell rupture [132,141]. In this mechanism, protein kinase C (PKC) activation, leads to NADPH oxidase (NOX) complex activation within endosomes and reactive oxygen species (ROS) production [142,143,144]. TcdB-mediated calcium release induces PKC activation, initiating multiple intracellular pathways [145]. ROS production results in ATP depletion, rapid lactate dehydrogenase (LDH) release, loss of caspase-3/7 activation, plasma membrane integrity loss, and chromatin condensation [141,146]. These events lead to cellular necrosis, potentially caused by DNA damage, protein oxidation, lipid peroxidation, and mitochondrial dysfunction. Unlike TcdB, TcdA does not induce lethal cell death but causes mucosal damage via glycosylation-dependent mechanisms at all concentrations. ROS levels from TcdA-induced mitochondrial damage are lower than those from NOX activation by TcdB, potentially explaining TcdA’s inability to cause lethal death [76,139,147].

Autophagy has been proposed as a mechanism of CDI induced by TcdB in a manner dependent on glycosylation and contributes to the inhibition of cell proliferation. Autophagy, a conserved intracellular degradation process, can enhance cytotoxicity and lead to cell death if prolonged. Even low TcdB concentrations increase the autophagy marker microtubule-associated proteins 1A/1B light chain 3B-II (LC3B-II), indicating autophagy induction. TcdB enhances phosphatidylinositol-3 kinase (PI3K) complex formation, crucial for autophagy initiation, and inhibits mammalian target of rapamycin complex 1 (mTOR) levels, boosting autophagy. The increase in autophagy depends on the action of the glycosyltransferase; however, the mechanism by which the enzymatic components directly induce host cell autophagy remains unclear [21,148].

Pyroptosis is an inflammatory type of programmed cell death triggered by infection, facilitated by TcdA and TcdB, and primarily affecting the host’s monocytes and macrophages. It is characterized by cell swelling and rupture of the plasma membrane, resulting in the release of cellular contents such as pro-inflammatory cytokines and danger-associated molecular patterns (DAMPs), triggering inflammation and recruiting immune cells to the site of cell death. Pyroptosis is mediated by a group of proteins called inflammasomes, which are a combination of protein complexes that activate inflammatory caspases, especially caspase 1. Activated caspase-1 leads to the activation of gasdermin proteins, ultimately resulting in plasma membrane pore formation, lysis, and release of cellular contents, fostering intense inflammation [149,150,151].

### 2.3. Interaction of TcdA and TcdB with the Host Immune Cells

Toxins can activate various intracellular signaling pathways responsible for the production and release of inflammatory mediators. The inflammatory response observed in CDI originates from the effects of toxins on the intestinal epithelial cells and is primarily driven by the activation of the innate immune system. Chemokines released recruit neutrophils and monocytes to the colonic lamina propria [34], resulting in fluid secretion and damage to the epithelium [61]. The inflammatory response is necessary to combat bacterial infection; however, when it escapes strict control, it can lead to extensive collateral tissue damage and contribute to the pathogenesis of CDI. It is worth noting that in clinical practice, the extent of inflammation observed in CDI, but not toxin levels, is the main prognostic indicator of poor CDI outcomes [152,153]. However, the mechanism behind the regulation of the proinflammatory activity of TcdA and TcdB remains unclear [154].

**Figure 3:**
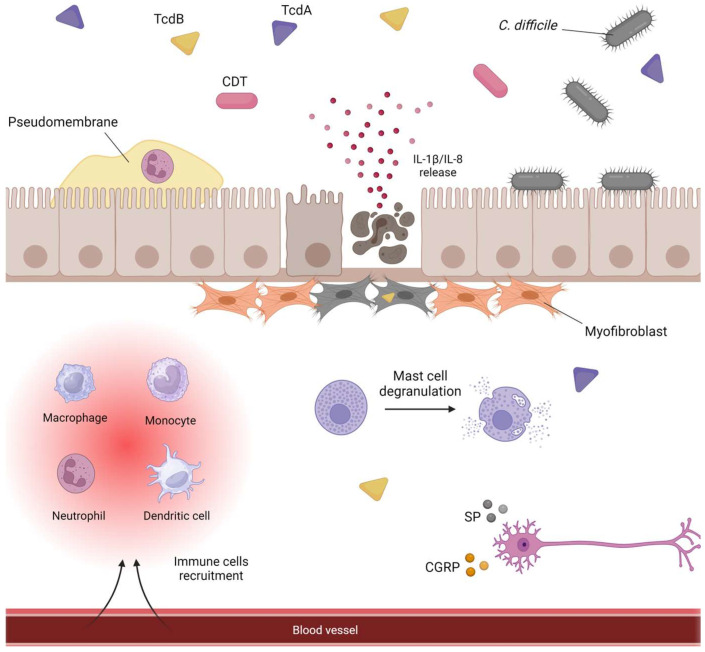
Representation of CDI-mediated inflammatory host response. Once TcdA and TcdB destroy the intestinal epithelium, they cause damage to deeper layers of tissue such as the destruction of the myofibroblasts. The presence of toxins triggers the release of dendritic cells, neutrophils, monocytes, and macrophages from the blood vessels. IL-1β and IL-8 produced by the intestinal epithelial cells enhance inflammation and attract neutrophils to the lumen of the colon. Within, neutrophils form pseudomembranes. At the same time, toxins induce the degranulation of mast cells and the release of substance P (SP) and Calcitonin gene-related peptide (CGRP) from neurons of the enteric nervous system.

TcdA and TcdB induce the release of inflammatory mediators from colonic epithelial cells, including interleukin-8 (IL-8) and interleukin-1 beta (IL-1β), correlating with CDI severity (Figure 3). IL-8 secretion, driven by toxins, attracts neutrophils into the colonic lamina propria [152,155,156]. Other released mediators include monocyte chemoattractant protein-1 (MCP-1) and interleukin-6 (IL-6) [31]. TcdA stimulates cyclooxygenase-2 (COX-2) production via the p38 mitogen-activated protein kinase (MAPK)/mitogen- and stress-activated protein kinase (MSK-1)/cAMP response element binding protein (CREB)/activating transcription factor-1 (ATF-1) pathway, leading to prostaglandin E2 (PGE2) secretion, which promotes fluid secretion in TcdA-induced enteritis [157]. Additionally, TcdA triggers C-X3-C motif chemokine ligand 1 (CX3CL1) secretion through p38 MAPK, IkB kinase (IKK), and nuclear factor kappa-light-chain-enhancer of activated B cells (NF-κB) activation in epithelial cells [158].

After the epithelial barrier disruption, TcdA and TcdB interact with monocytes and macrophages, stimulating high levels of IL-8, IL-1β, and tumor necrosis factor-alpha (TNF-α) secretion [155,159,160]. These factors recruit neutrophils to the colonic lamina propria [161]. Toxins induce IL-8 and IL-1β secretion via early and sustained p38 MAP kinase activation, independent of glycosylation, and inflammasome activation, particularly the pyrin inflammasome [153,162,163]. Pyrin is the product of the Mediterranean fever gene and acts as an intracellular sensor responding to glycosylated Rho GTPases, mediating glycosylation-dependent activation of the inflammasome [164]. Despite both toxins’ ability to activate the inflammasome, TcdB induces activation more potently, even at much lower concentrations than TcdA [165]. Inflammasome activation involves two steps: priming, where NF-κB induces pro-IL-1β production in response to danger signals, and activation, where the inflammasome assembly activates caspase-1 [166,167]. Caspase-1 then processes pro-IL-1β into IL-1β, triggering a cascade leading to the release of interleukin-18 (IL-18) and gasdermin D. These cytokines stimulate the release of IL-6, interferon-gamma (IFN-γ), and IL-8 from the epithelial cells of the colon [160,168]. Caspase-1 cleaves gasdermin D, releasing its N-terminal end, which inserts into the plasma membrane, forming pores and causing osmotic swelling, resulting in pyroptotic cell death [100].

TcdA and TcdB stimulate dendritic cells to produce TNF-α directly [169]. IL-1β from inflammasome activation enhances interleukin 23 (IL-23) production by dendritic cells [170]. Elevated IL-23 levels in CDI recruit neutrophils to the colon, mediating inflammation. TcdA and TcdB, in conjunction with MyD88-dependent danger signals, boost IL-23 production by increasing IL-1 secretion and subsequent signaling [171]. TcdB also activates group 3 innate lymphoid cells (ILC3s) directly by glycosylating Cdc42. ILC3s, found in the gastrointestinal mucosa, defend against pathogens and are activated by IL-23 and IL-1β to produce Interleukin-22 (IL-22). IL-22 in CDI models regulates microbiota metabolism, induces antimicrobial peptides, and activates the complement system, providing protective effects [172].

TcdA and TcdB stimulate intestinal mast cells, crucial for the innate immune response against *C. difficile* toxins. Mast cell activation by toxins induces degranulation and release of IL-8, TNF-α, and histamine. Toxins weaken granule cohesion with the plasma membrane, facilitating degranulation via actin rearrangement. TcdB activates intracellular signaling in mast cells, including p38 MAP, promoting prostaglandin production and degranulation [138,173]. Interestingly, mast cell-deficient mice show reduced inflammatory responses and do not develop TcdA-induced enteritis [153].

Colon epithelial cytokines activate enteric sensory neurons [173]. TcdA induces inflammation by releasing neuropeptides like Substance P (SP) and Calcitonin gene-related peptide (CGRP) from sensory neurons, enhancing the inflammatory signal [174,175] (Figure 3). SP stimulates intestinal macrophages in lamina propria, contributing to TNF-α release and epithelial cell destruction. TcdA exposure increases the SP receptor neurokinin-1 expression on epithelial cells. Increased expression of neurokinin-1 has been detected in the intestines of patients with pseudomembranous colitis, highlighting its role in the pathogenesis of CDI [175]. Notably, mice lacking neurokinin-1 show reduced intestinal responses to TcdA [176].

### 2.4. Toxemia and Extraintestinal Damage Caused by TcdA and TcdB

**Figure 4:**
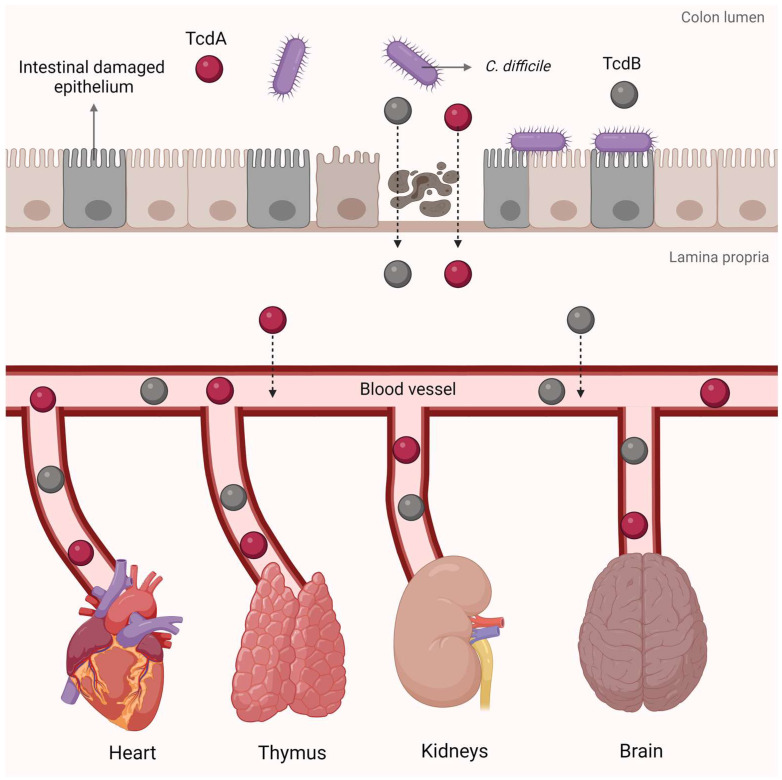
During CDI, *C. difficile* toxins TcdA and TcdB can breach the intestinal barrier and enter the bloodstream, resulting in systemic toxemia. Elevated levels of these toxins in the bloodstream can cause damage outside the colon, leading to dysfunction in multiple organs such as the heart, thymus, kidneys, and brain.

While CDI primarily affects the colon, it can precipitate systemic complications [177,178] (Figure 4). Animal studies have demonstrated manifestations such as cardiopulmonary injury, ascites, organ failure, and acute respiratory distress syndrome [179,180]. In pigs and mice infected with BI/NAP1/027 spores, high toxin levels have been detected in their sera, pleural, and ascitic fluids. Toxin presence in systemic circulation directly correlated with systemic CDI manifestations, absent in animals without systemic complications. Similarly, patients suffering from severe CDI experience similar systemic effects, including multiple organ dysfunction and soft tissue infections [178,179].

Unlike most cases of bacterial sepsis, the organ damage inflicted by CDI likely results from toxemia. Both TcdA and TcdB can breach the intestinal barrier, causing systemic toxemia in animal models [171,179]. Toxins can also increase colon vascular permeability via vascular endothelial growth factor A (VEGF-A) production, observed in human colonic mucosa biopsies and serum of CDI patients. *C. difficile* strains producing TcdA and/or TcdB induce VEGF-A in mice with CDI, promoting a more permeable vascular barrier and explaining systemic toxin spread in CDI animal models and human toxemia cases [181].

Notably, pediatric cases of fatal pseudomembranous colitis have reported the presence of cytotoxins in serum and ascitic fluid, often in the context of underlying conditions [182]. In 2021, researchers found that patients with severe CDI had higher levels of serum TcdA (>60 pg/μL) compared to those with mild cases. They also observed toxemia in 33 out of 35 patients studied, indicating a high prevalence of toxins in the bloodstream. This suggests a potential association between elevated serum TcdA levels and the development of severe complications in CDI cases [183].

Although circulating TcdA and TcdB have been observed in CDI patients, further investigation is required to ascertain their correlation with extraintestinal damage [178]. The systemic impact of TcdB during CDI extends to lymphoid tissues such as the thymus. This observation is particularly pertinent for elderly patients, resulting in the weakening of the immune system, and leading to an increased risk of CDI recurrence or secondary infections [184]. There are indications that toxins may affect organs like the kidneys, brain, and heart (Figure 4). Research indicates that TcdA and TcdB can cause kidney damage through various mechanisms, including hindering healing, prompting apoptosis in renal epithelial cells, and decreasing renal perfusion pressure and glomerular filtration rate [185,186,187]. Furthermore, studies suggest that toxins induce apoptosis in cerebellar granule neurons by disrupting Rho GTPases, triggering a cascade of proapoptotic signaling disturbances [188,189]. In addition, a study on zebrafish reveals that toxins detrimentally affect the heart, leading to reduced heart rate, structural deformities, decreased cardiomyocyte viability, and pericardial edema [190].

## 3. *C. difficile* Transferase (CDT)

There are some *C. difficile* strains, such as the BI/NAP1/027 strain, that can also produce the binary toxin CDT [37,40]. CDT was first isolated from strain CD196 (RT 027) from a patient with severe pseudomembranous colitis [191,192]. It is produced by 5–30% of clinical isolates of *C. difficile* and is associated with more severe complications, longer hospitalization, and increased mortality rates [193,194,195,196]. The production of CDT has also been correlated with increased antibiotic resistance, and the detection of its gene could be used as a marker for antimicrobial susceptibility [197]. CDT belongs to the family of binary ADP-ribosylating toxins [198]. Other members of this group include the C2 toxin of *Clostridium botulinum*, the iota toxin of *C. perfringens*, the Vegetative Insecticidal Protein (VIP) of *Bacillus cereus*, *Clostridium spiroforme* toxin (CST), and the lethal toxins of *Bacillus anthracis*. In contrast to LCTs that enter host cells as single polypeptides, binary toxins are secreted by the bacterium as two separate components A and B, and enter cells after binding component B to their receptors [73,199].

### 3.1. Genetics and Structure of CDT

**Figure 5:**
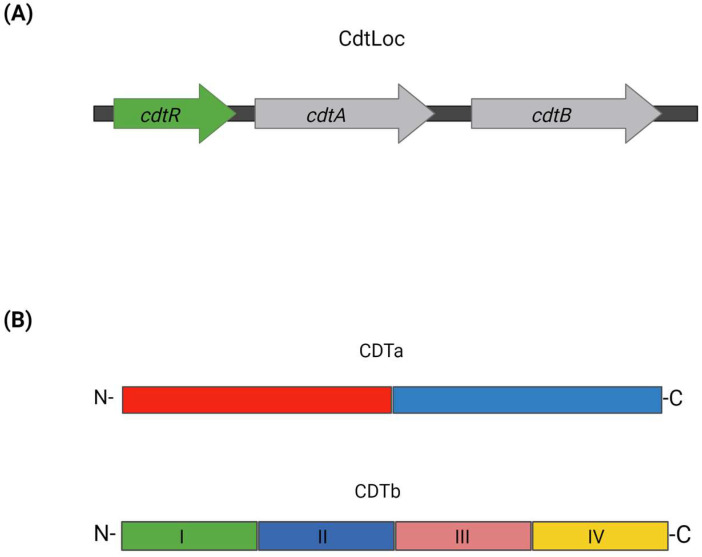
(**A**) Representation of CDT locus (CdtLoc). Genes *cdtA* and *cdtB* (grey arrows) encode the components CDTa and CDTb, respectively. The transcription of *cdtA* and *cdtB* is positively regulated by the regulatory gene *cdtR* (green arrow). The direction of the arrows represents the direction of transcription of the genes. (**B**) Schematic representation of CDTa and CDTb components of CDT. CDTa is divided into two regions: the N-terminal region and the C-terminal region. CDTb is divided into four conserved functional regions (Regions I–IV).

CDT consists of two components, CDTa (enzymatic component) and CDTb (binding component) [200]. The genes encoding CDT are located in a 6.2 kb genetic region, distinct from the PaLoc, known as the CDT locus or CdtLoc [11]. In many *C. difficile* strains that do not produce CDT, the CdtLoc is replaced by a conserved sequence of 68 bp length. The CdtLoc includes the genes *cdtA* and *cdtB* encoding the components CDTa and CDTb, respectively, and *cdtR* encoding the protein CdtR [40] (Figure 5). The CdtR protein is a positive transcriptional regulator belonging to the LytTR family, and its role is to activate the production of CDT [11]. The secretion mechanism of CDT is not well-known, as unlike PaLoc, CdtLoc lacks genes associated with transport and pore formation mechanisms [43,201].

CDT comprises CDTa and CDTb [22,202] (Figure 5). CDTa is a 53 kDa protein with 463 amino acids, consisting of N- and C-terminal regions [203,204]. The N-terminal undergoes proteolytic cleavage, resulting in a mature form (~48 kDa) [198,201]. It shares homology with *C. perfringens* iota toxin and *C. spiroforme* toxin [205]. The N-terminal (residues 1–215) of mature CDTa interacts with CDTb, while the C-terminal (residues 224–420) harbors ADP-ribosyltransferase activity [205,206]. CDTb, the binding component, has a molecular mass of 99 kDa and consists of 876 amino acids [201,203]. It shares homology with binding components of other toxins such as iota toxin (Ib, 77%) and CSTb (77%) of *C. spiroforme* toxin. CDTb is divided into four functional regions: activation domain I, pore formation region II, oligomerization region III, and membrane receptor binding region IV [73,205]. Proteolytic cleavage by serine proteases yields a mature protein [203]. Activation of CDTb involves cleavage of the region I activation, releasing a ~20 kDa propeptide, followed by oligomerization to form activated CDTb. Cleavage of the chain occurs between Lys209 and Leu210 of the preform [198].

### 3.2. Mode of Action of CDT

**Figure 6:**
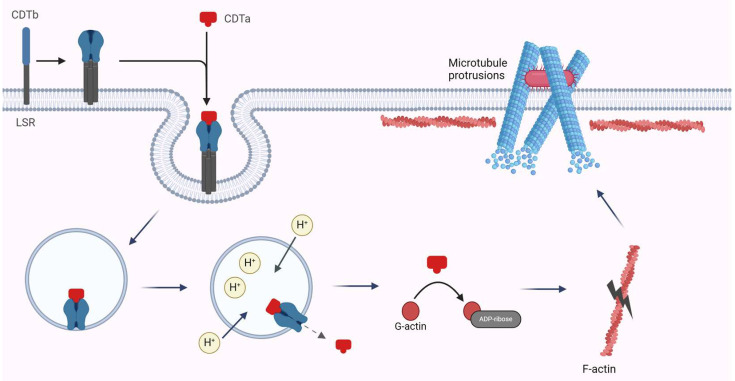
Schematic representation of CDT binding to cellular receptor and entry into the cell. CDTb binds into LSR and oligomerizes on the cell surface. Therefore, CDTa binds to the oligomeric form, and the complex is internalized into cells. The acidic environment of endosomes triggers conformational changes in CDTb resulting in a pore formation in the endosomal membrane and the translocation of CDTa into the cytosol. CDTa catalyzes the ADP-ribosylation of actin resulting in the disruption of F-actin and the formation of microtubule protrusions.

Cellular intoxication by CDT occurs in three stages: (i) binding of the toxin to cell surface receptors; (ii) cellular uptake via endocytosis and formation of pores in the endosomal membrane; and (iii) translocation of the toxin into the cytosol and ADP-ribosylation of actin and microtubule protrusion formation (Figure 6).

#### 3.2.1. Binding to Cellular Receptors

CDTb binds to lipolysis-stimulated lipoprotein receptor (LSR) on the cell surface [201,207]. LSR is a type I transmembrane protein with an extracellular immunoglobulin-like domain and an intracellular domain [198]. Precursor CDTb interacts with LSR via residues 653–834 [208]. It has been shown that LSR acts as a receptor for iota toxin and CST [207]. Iota toxin’s Ib and CST’s Sb share 91% and 89% homology with precursor CDTb, respectively, suggesting conserved transport function [76]. LSR is expressed in various tissues, including the liver, lungs, intestines, and kidneys. Its functions include triglyceride-rich lipoprotein uptake, low-density lipoprotein (LDL) clearance, and the formation of TJs in epithelial cells [73,198]. CD44, another cell surface protein, can also act as a receptor for clostridial toxins. Mice lacking the CD44 gene exhibit partial toxin resistance, suggesting its potential role as a co-receptor, although specifics remain unclear [209].

#### 3.2.2. Cellular Uptake and Pore Formation

Once CDTb binds to LSR, it undergoes proteolytic cleavage, inducing clustering of LSR in lipid rafts [210]. This promotes oligomerization into a heptamer on the cell surface [43]. Proteolysis is crucial for oligomerization and subsequent cellular entry [203]. CDTa binds to the heptamer, facilitating endocytosis into acidic endosomes [211]. Conformational changes in CDTb at low pH lead to pore formation in the endosomal membrane, allowing translocation of CDTa into the cytoplasm [212]. Host proteins, including heat shock proteins 70 and 90, FK506-binding protein, and cyclophilin A, are implicated in this translocation process [213,214].

The production of CDTb alone is sufficient to enhance the pathogenic action of the bacterium [215]. CDTb exhibits cytotoxic activity in cultured Caco-2 and Vero cells in the absence of CDTa. CDTb induces cell rounding, impairs their viability, and disrupts the epithelial integrity of cultured Caco-2 monolayers in a manner dependent on the binding of CDTb to the LSR receptor. Blocking LSR with inactive CDTa and a pore inhibitor protected cells from CDTb-induced cytotoxicity, confirming that pore formation in the cell membrane by CDTb is responsible for its cytotoxic effects [216].

#### 3.2.3. ADP-Ribosylation of Actin and Microtubule Protrusion Formation

Inside the cell, CDTa alters the structure of monomeric G-actin through ADP-ribosylation at arginine-177 [40,201]. Mammals possess six tissue-specific isoforms of actin, including skeletal α-actins, cardiac α-actins, α- and γ-smooth muscle actins, and β- and γ-cytoplasmic actins. CDT appears to modify most, if not all, actin isoforms. Normally, monomeric G-actin polymerizes to form filaments of F-actin. Polymerization occurs at the rapidly growing positive or barbed end. Upon incorporation of G-actin into the positive end of the growing F-actin, the ATP bound to G-actin is hydrolyzed to ADP and inorganic phosphate. Polymerization continues until equilibrium is reached between G- and F-actin. Conversely, depolymerization occurs at the negative or pointed end, from which actin monomers are dissociated [205].

CDT introduces ADP-ribose onto arginine 177 of G-actin, disrupting the F-actin structure and terminating its polymerization [19,217]. Ribosylated G-actin interacts exclusively with the positive end and inhibits polymerization. Simultaneously, the unmodified negative end of F-actin continues to depolymerize, ultimately leading to a complete breakdown of the actin cytoskeleton [205]. This modification prevents the normal assembly of actin filaments, resulting in cell rounding, disruption of TJs, and disturbances in various cellular activities dependent on actin polymerization, such as cell motility, phagocytosis, endocytosis, and secretion [201].

CDT’s impact on epithelial cells includes disrupting microtubule organization and dynamics [218]. Microtubules consist of α-/β-tubulin heterodimers and have a polarized structure [219]. Their growth toward the cell membrane is impeded by cortical actin beneath the membrane [73]. CDT disrupts cortical actin, leading to elongated membrane protrusions fed by uncontrolled microtubule growth [220]. These protrusions, enriched with endoplasmic reticulum tubules, form a network allowing bidirectional movement of vesicles. Bacteria adhere to these abnormal protrusions, enhancing colonization [221].

Protrusion formation relies on cholesterol and sphingolipid-rich lipid microdomains in the membrane. Lowering cholesterol reduces protrusion number, while cholesterol replenishment boosts their formation. Sphingolipid presence is also crucial; inhibiting their biosynthesis reduces protrusions. Increasing membrane fluidity and disrupting lipid rafts inhibit protrusion development. The mechanism of protrusion formation involves F-actin depolymerization, leading to a redistribution of microtubule-stabilizing proteins like actin crosslinking family protein 7 (ACF7) and cytoplasmic linker-associated protein 1 (CLASP) [222,223]. This redistribution likely impairs microtubule guidance [205]. Septins, associated with microtubules, actin, and membranes, also play a crucial role [43]. CDT-induced F-actin depolymerization triggers septin redistribution to the membrane, where they organize into ring-like structures, guiding microtubule growth through interactions with proteins like the end binding 1 (EB1) and the end binding 3 (EB3) [205,223]. F-actin depolymerization redirects fibronectin from the basolateral to the apical membrane, aiding bacterial adherence. Microtubule-guided vesicle movement to the apical region releases adhesive glycoproteins, further enhancing bacterial adhesion [73].

### 3.3. Interaction of CDT with the Host Immune Cells

CDT induces an increased local and systemic inflammatory response in the host and enhances the disruption of the host defense mechanisms caused by TcdA and TcdB. CDT triggers an inflammatory response in the host by suppressing the protective eosinophils of the colon and blood through the indirect induction of eosinophil apoptosis. When CDT acts synergistically with TcdA and TcdB, it can activate the transcription factor NF-κB and increase the production of IL-1β. The induced production of IL-1β by cells of the innate immune system depends on signaling through Toll-like receptor 2 (TLR2) and Toll-like receptor 4 (TLR4) [224,225]. TLR2 requires heterodimerization with Toll-like receptor 6 (TLR6) for the heterodimeric TLR2/6 to recognize CDT and activate signaling cascades [226]. The binding of CDT to TLR2 and TLR4 receptors on macrophages may also induce the secretion of chemokine (C-X-C motif) ligand 2 (CXCL2) and TNF-α [225]. The CDTb component activates human mucosal-associated invariant T (MAIT) cells associated with mucosal immunity. Activated MAIT cells mediate cytotoxicity by releasing lytic granules containing cytotoxic molecules. Activation of MAIT by CDT depends on interleukin-19 (IL-19) and the MRI-dependent signaling pathway [227].

## 4. Therapeutic Strategies Based on Toxins

CDI is typically treated with antibiotics, with vancomycin, metronidazole, and fidaxomicin being the recommended choices for both primary and recurrent cases. However, the prolonged use of antibiotics may cause antibiotic resistance and microbiota disruption, which predispose to recurrence. These limitations highlight the need for novel and effective management strategies [228]. Contemporary therapeutic approaches to CDI primarily focus on targeting the toxins produced by *C. difficile*. Research interest has therefore centered on developing new therapies that aim to neutralize these toxins, addressing the effects of toxins that are not tackled by current antibiotic-based treatment regimens. Toxin-based therapies not only help in treating the infection but also promote the preservation of the host’s microbiota. Additionally, vaccination against toxins offers protection against CDI by activating the immune system to defend against the development of the infection [23,76,229].

### 4.1. Antibody-Based Therapies

Human monoclonal antibodies, including actoxumab and bezlotoxumab, show promise in neutralizing TcdA and TcdB associated with CDI. As of now, bezlotoxumab stands as the sole additional anti-toxin therapy that has been FDA-approved for preventing CDI recurrence in high-risk adults undergoing antibiotic treatment [230,231]. Bezlotoxumab is an IgG1 monoclonal antibody that targets the CROPs domain of TcdB by binding to two adjacent epitopes within this domain and effectively disrupts the interaction between TcdB and CSPG4. It is administered intravenously alongside antibacterial therapy, and likely reaches the intestinal lumen through paracellular transport facilitated by toxin-induced epithelial barrier disruption. This mechanism suggests its potential efficacy in treating severe CDI episodes [232]. Despite its clinical use, bezlotoxumab has limitations because it is unable to interfere with the interaction between TcdB and FZD1,2,7 and PVRL3 receptors [74,233]. Furthermore, due to mutations in the TcdB epitopes of hypervirulent *C. difficile* strains, bezlotoxumab’s neutralizing activity may be restricted [23].

Actoxumab, a monoclonal antibody targeting repeats within the CROPs domain of TcdA to block its binding, showed limited efficacy in clinical trials [230,231]. Despite effectively neutralizing TcdA in vitro across various clinical isolates when used alone, the transition of clinical trials from phase I to phase II was halted due to observed ineffectiveness. Actoxumab’s efficacy limitation may arise from its partial blocking of only two out of seven carbohydrate bindings sited in TcdA’s CROPs domain, impairing receptor binding. Unlike bezlotoxumab, it cannot simultaneously bind both epitopes, resulting in less impact on toxin conformation. Additionally, other regions influencing TcdA entry into cells and a variety of toxin strains may impact neutralization potency for both actoxumab and bezlotoxumab [23].

Exploring antibodies targeting different TcdB positions may broaden therapeutic options for CDI. Various antibodies have been reported in experimental studies. The neutralizing antibody 5D targets the DRBD domain of TcdB, inhibiting pH-induced conformational changes necessary for pore formation. The neutralizing antibody 7F targets the C-terminal end of the GTD domain, inhibiting GTD proteolytic cleavage. E3 antibody targets GTD, inhibiting glycosylation [234]. PA41 antibody targets a GTD epitope, preventing GTD translocation into the host cell cytoplasm [60]. Antibodies 5D and E3, when genetically fused with antibodies against TcdA in a *Saccharomyces boulardii* strain, administered as a probiotic, effectively combat CDI in mice [235]. Designed ankyrin repeat proteins (DARPins) hinder TcdB’s interaction with CSPG4 and FZD2 [81].

### 4.2. Antimicrobial Peptides

Antimicrobial peptides, including α-defensins 1 and 5, neutralize the effects of toxins like TcdA, TcdB, and CDT. These peptides are small, cationic molecules, expressed mainly in neutrophils and Paneth cells of the small intestine. α-defensins are crucial components of innate immune defense combating pathogenic bacteria and various bacterial toxins. Upon encountering toxins like TcdA, TcdB, and CDT, α-defensins exert their antimicrobial activity by disrupting toxin function and preventing their effects on host cells. One notable mechanism by which α-defensins neutralize toxin activity is through the inhibition of pore formation by CDTb. By interfering with the assembly or stability of the CDTb pore, α-defensins effectively block the cytotoxic effects of CDT, thereby protecting host cells from damage [236,237,238].

### 4.3. Pharmacological Inhibitors

CDT interacts with certain cellular proteins that help the transport of CDTa from endosomes to the cytoplasm, including Hsp90, Hsp70, and peptidyl-prolyl cis/trans isomerases of cyclophilin (Cyp), and FK506-binding protein (FKBP) families, blocking these proteins can shield cells from CDT’s toxicity. Pharmacological inhibitors like Radicol and VER-155008 can inhibit the activity of Hsp90 and Hsp70, respectively. Also, Cyclosporine A and FK506 inhibit the activity of Cyps and FKBPs, respectively. The combination of these inhibitors has shown a strong ability to block CDT intoxication in cells [239]. Additionally, chloroquine and its derivatives can protect the HCT 116, Vero, and Caco-2 cells from CDTb intoxication, inhibit CDTb pore formation, and prevent the cytotoxic effects of the CDTa and CDTb combined action [212].

### 4.4. Small Molecule Inhibitors

Some small inhibitors block the enzymatic properties of toxins and the completion of autoproteolytic processing. The molecule ebselen is being investigated both for inhibiting GTD function and for inhibiting APD action simultaneously. Ebselen reduced inflammation and CDI recurrence rates, protected hamsters from tissue damage, and enhanced gut microbiota recovery in mice following antibiotic treatment [240]. Additionally, it has been reported that this molecule can inhibit NADPH oxidase activity, thus suppressing ROS production [241]. Similarly, N-acetylcysteine, an FDA-approved antioxidant, can act in the final stage of cellular necrosis, after ROS generation, and prevent tissue damage caused by TcdB [143]. Moreover, calcium channel blockers with a dihydropyridine nucleus can interfere with calcium signaling induced by TcdB and reduce ROS production [145]. Niclosamide inhibits endosomal acidification by increasing pH. Niclosamide protects cells from the effects of all three toxins, which require a pH drop for pore formation and entry into the host cytoplasm, even from the TcdB of hypervirulent strains, which undergo conformational changes at higher pH. Treatment of mice with CDI improved symptoms of primary infection and recurrence without affecting the intestinal microbiota [242].

### 4.5. Vaccination

The ability of the immune response and the presence of circulating antibodies against TcdA and TcdB have been associated with host protection against severe or recurrent CDI [243,244]. Clinical studies focused on developing vaccines based on inactivated toxins (toxoids) and recombinant toxins to elicit systemic antibody responses against TcdA and TcdB [245]. Three CDI vaccines are in clinical trials. The first vaccine, developed by Sanofi Pasteur, used formalin-inactivated full-length toxins TcdA and TcdB, but the vaccine’s development was halted after phase III clinical trials. The second vaccine, developed by Pfizer, was based on full-length recombinant molecules of TcdA and TcdB. The vaccine was tested in phase III clinical trials and showed potential in reducing the severity of CDI, although it did not prevent initial infection. These results demonstrated that a vaccine containing only toxoids cannot prevent bacterial transmission and initial infection. The third vaccine (research name VLA84), developed by Valneva Austria GmbH, is based on the use of a single recombinant fusion protein consisting of segments from the C-terminal ends of TcdA and TcdB. VLA84 completed phase II clinical trials [246].

## 5. Conclusions

CDI is one of the most serious and immediate healthcare-associated infections and poses substantial challenges to patient care and healthcare systems worldwide. Over the last decade, the epidemiology of CDI has evolved, with an increase in disease incidence and severity [247]. The clinical symptoms of CDI result from the action of toxins TcdA, TcdB, and CDT. These toxins consist of functional domains that allow them to interact with host cells, enter them, and induce cellular damage [248]. While progress has been made in understanding their mechanisms, further exploration is needed.

Continuing research aims to identify new receptors and toxin domains, particularly focusing on understanding how TcdB variants interact with cellular receptors. This exploration could offer insights into developing targeted therapies for epidemic strains like BI/NAP/027.

Furthermore, the delivery mechanism of the effector domains of TcdA and TcdB into the host cytosol is still unclear. Once there, these domains disrupt cellular processes, contributing to disease pathology. Understanding pore formation and translocation in the host cytosol is a key research focus.

Future studies could focus on identifying intracellular targets of TcdB variants from different *C. difficile* strains and their impact on CDI severity. Investigating specific GTPase targets glycosylated by these variants could reveal insights into disrupted intracellular processes. Understanding their specificity in targeting host cell pathways may unveil new therapeutic targets for CDI treatments.

While CDT damages the epithelial tissue by disrupting the actin cytoskeleton and inducing inflammation, its role in CDI is less understood compared to TcdA and TcdB. Further study is needed to elucidate its impact on severe infection outcomes.

CDI’s impact extends beyond the gastrointestinal tract to affect vital organs. This suggests the occurrence of systemic toxemia, wherein toxins spread throughout the body, potentially influencing the prognosis of CDI patients. Understanding these highlights is crucial for comprehensive management strategies beyond targeting gut-specific symptoms.

Despite extensive pre-clinical investigations into alternative toxin-targeting therapeutic strategies, only bezlotoxumab and actoxumab have progressed to clinical trials, emphasizing the need for further treatment research. Understanding how TcdB mutations affect the efficacy of neutralizing agents like bezlotoxumab is crucial for developing effective treatments. Furthermore, the investigation of the structural and functional characteristics of toxins is vital for designing therapeutic antibodies and vaccines.

In conclusion, while significant progress has been made in understanding the role of toxins in CDI pathogenesis, the further understanding of virulence factors, pathogenicity, and host interactions will aid the development of novel alternative therapeutics. Continued research in this area will be critical for improving outcomes for patients with CDI and reducing the burden of this challenging infection on healthcare systems worldwide.

## Data Availability

No new data were created or analyzed in this study. Data sharing is not applicable to this article.

## Abbreviation

APD	Autoprotease domain
CDI	*Clostridioides difficile* infection
CDK1	Cyclin-dependent kinase 1
CDT	*Clostridioides difficile* transferase
CDTa	*Clostridioides difficile* transferase component a
CDTb	*Clostridioides difficile* transferase component b
CdtLoc	*Clostridioides difficile* transferase locus
CGRP	Calcitonin gene-related peptide
CPE	Cytopathic effect
CROPs	Combined Repetitive Oligopeptides
CSPG4	Chondroitin Sulfated Proteoglycan 4
DRA	Down-regulated in adenoma
DRBD	Delivery/Receptor Binding Domain
EGCs	Enteric Glial Cells
FZDs	Frizzled Receptors
gp96	Glycoprotein 96
GDP	Guanosine diphosphate
GTD	Glycosyltransferase Domain
GTP	Guanosine-5′-triphosphate
GTPases	Enzymes associated with guanosine triphosphate
IL-1β	Interleukin-1 beta
IL-6	Interleukin-6
IL-8	Interleukin-8
LCTs	Large clostridial toxins
LDLR	Low-density lipoprotein receptor
LRP1	Receptor-Related Protein 1
LSR	Lipolysis-stimulated lipoprotein receptor
MOMP	Mitochondrial outer membrane permeability
NHE3	Sodium-hydrogen Exchanger 3
PACSIN2	Protein kinase C and the substrate for casein kinase 2
PaLoc	Pathogenicity locus
PKC	Protein kinase C
PVRL3	Poliovirus Receptor-Like 3
ROS	Reactive oxygen species
sGAGs	Sulfated Glycosaminoglycans
SP	Substance P
TcdA	*Clostridioides difficile* toxin A
TcdB	*Clostridioides difficile* toxin B
TFPI	Tissue Factor Pathway Inhibitor
TJ	Tight junction
TLR	Toll-like receptor
TNF-α	Tumor necrosis factor-alpha
VEGF-A	Vascular endothelial growth factor A
ZO	Zonula occludens
